# Exploring the advection-diffusion equation through the subdivision collocation method: a numerical study

**DOI:** 10.1038/s41598-024-52059-7

**Published:** 2024-01-19

**Authors:** Safia Malik, Syeda Tehmina Ejaz, Ali Akgül, Murad Khan Hassani

**Affiliations:** 1 Department of Mathematics, The Government Sadiq College Women University Bahawalpur, Bahawalpur, 63100 Pakistan; 2 Department of Computer Science and Mathematics, Lebanese American University, Beirut, Lebanon; 3 Art and Science Faculty, Department of Mathematics, Siirt University, Siirt, 56100 Turkey; 4 Mathematics Research Center, Department of Mathematics, Near East University, Near East Boulevard, Nicosia/Mersin 10, 99138 Turkey; 5 Department of Mathematics, Ghazni University, Ghazni, Afghanistan

**Keywords:** Engineering, Mathematics and computing, Physics

## Abstract

The current research presents a novel technique for numerically solving the one-dimensional advection-diffusion equation. This approach utilizes subdivision scheme based collocation method to interpolate the space dimension along with the finite difference method for the time derivative. The proposed technique is examined on a variety of problems and the obtained results are presented both quantitatively in tables and visually in figures. Additionally, a comparative analysis is conducted between the numerical outcomes of the proposed technique with previously published methods to validate the correctness and accuracy of the current approach. The primary objective of this research is to investigate the application of subdivision schemes in the fields of physical sciences and engineering. Our approach involves transforming the problem into a set of algebraic equations.

## Introduction

The Advection Diffusion Equation (ADE) is a significant mathematical model with crucial applications in various industrial, scientific, and engineering fields. It elucidates how substances or quantities disperse within a fluid medium. Prominent applications encompass environmental modeling, fluid dynamics, heat transfer, atmospheric science, pharmacokinetics, and geological processes. In several physical phenomena, the Advection Diffusion Equation delineates the combined effects of advection (bulk flow transport) and diffusion (random spreading). In engineering, it finds utility in simulating the transfer of substances like heat, contaminants, or solutes within fluids. Advection characterizes bulk movement, while diffusion accounts for the spreading driven by concentration gradients. This equation plays a pivotal role in comprehending and predicting substance dispersion in engineering applications, such as fluid dynamics, heat transfer, and pollution dispersion. Consider one-dimensional advection diffusion transport equation of the form

1 $$\begin{aligned}{} & {} \frac{\partial u(x,t)}{\partial t}+\beta \frac{\partial u(x,t)}{\partial x} =\alpha \frac{\partial ^{2} u(x,t)}{\partial x^2}, \,\, (x,t)\in [0,L]\times [0,T] \end{aligned}$$

2 $$\begin{aligned}{} & {} u(x,0)=\psi _{1}(x), \end{aligned}$$

3 $$\begin{aligned}{} & {} u(0,t)=\phi _{1}(t), \,\ u(L,t)=\phi _{2}(t), \,\ t\in [0,T] \end{aligned}$$

with the initial condition in (2) and boundary conditions as stated in (3). Here, $\beta$ represents the velocity of the flow, and $\alpha$ is the diffusion coefficient. The concentration at the point *x* and at time *t*, denoted as *u*(*x*, *t*), is an unknown function. The length of the channel is denoted by *L*, and $\psi _1(x)$, $\phi _{1}(t)$, and $\phi _{2}(t)$ are given functions. The governing equation (1) becomes a heat equation if the advection speed $\beta$ is zero with a positive diffusion coefficient. Also $\psi _1(x)$, $\phi _{1}(t)$ and $\phi _{2}(t)$ are the known smooth functions.

In the past, various authors have studied mathematical models from different perspectives. For instance, Mohebbi, A. and Dehghan, M., proposed a higher-order compact solution for the advection-diffusion equation (ADE) (1). They employed a finite difference approximation to discretize the spatial derivatives and developed the cubic $C^{1}$-spline collocation method to solve the resulting linear system of ordinary differential equations^1^. Additionally, new methods, such as cubic trigonometric B-spline, cubic B-spline, redefined cubic B-spline collocation, and exponential B-spline collocation, have been utilized to solve (1) in studies by^2–6^.

Numerical solution of Advection-Diffusion Equation using graph theoretic polynomial collocation method was presented in previously published work^7^. Numerical solution of advection-diffusion equation using a sixth-order compact finite difference method was given in^8^. The investigation of the Eq. (1) was conducted using B-spline functions. Equation (1) finds numerous physical applications, such as modeling rapid flows through porous surfaces, tracer dispersion, contaminant spreading in rivers and streams, and the dispersion of dissolved salts in groundwater and water transfer^9^. The combination of B-splines and Taylor–Galerkin techniques was used to approximate functions over finite elements for solving (1)^10^. Intermediate Dirichlet boundary conditions were formulated in^11^ for Strang-type splitting algorithms applied to the one-dimensional advection-diffusion equation. In^12^, an investigation about the reaction advection-diffusion equation with a variable intrinsic growth rate was presented using Robin and free boundary conditions. Differential quadrature method based on B-spline functions of degrees four and five was used to solve (1), numerically in^13^. Additionally, the time-dependent one-dimensional linear advection-diffusion equation with Dirichlet homogeneous boundary conditions and an initial sine function was solved analytically through separation of variables and numerically using the finite element method^14^. The computational modeling of coupled advection-diffusion-reaction systems was presented in^15^, while the Galerkin-finite element method was employed to solve (1) as demonstrated in^16^.

The purpose of this research work is to develop a mathematical models and subsequent numerical methods for (1), estimating pollution induced at different time and locations in water bodies using subdivision method.

Numerical algorithms based on subdivision schemes are not commonly employed for obtaining numerical solutions to boundary value problems (BVPs). Subdivision-based techniques have been instrumental in calculating approximate solutions to BVPs. Qu and Agarwal were the first to develop these algorithms^17–19^. Their approaches were based on an interpolatory subdivision algorithm for second-order two-point boundary value problems (BVPs). After a long gape, the subdivision collocation method has been used to solve ordinary differential equations with boundary conditions by^20–22^. Based on existing literature, subdivision collocation methods have not yet been applied to solve partial differential equations (PDEs). This numerical approach demonstrates rapid convergence, smoothness, and requires less computational time compared to existing methods. This motivated us to employ the subdivision collocation method to solve (1).

In this paper, our aim is to develop an efficient and convenient numerical approach for solving Eq. (1) with various advection and diffusion parameters, using different choices of time and space grid points. The results demonstrate the efficiency of this new technique when compared to existing methods^1–6,23,24^.

This research work is organized as follows: In the “Subdivision Scheme” section, it discusses some key characteristics of the six-point binary interpolating subdivision scheme. In the “Subdivision collocation method” section, a subdivision collocation method is developed, including the initial guess and iterative algorithm, for the solution of an ADE problem. The “Stability analysis and estimation of errors” section presents the results regarding the convergence of the present algorithm and the method for calculating errors. The “Numerical examples, results and discussion” section compares the findings based on the present algorithm with other existing approaches in the “Numerical examples, results and discussion” section. The last section presents the conclusions.

## Subdivision scheme

Subdivision schemes are efficient tools for generating smooth curves and surfaces efficiently from a discrete set of control points. This is consistent and efficient iterative algorithm to be used for modeling of curve and surfaces. Subdivision schemes can be classified into two important branches such as approximating and interpolating. In the approximating scheme, the limit curve approximates the initial polygon, and after subdivision, only the newly generated control points are part of the limit curve. In the interpolating scheme, both the control points of the original control polygon and the newly generated control points lie on the limit curve after subdivision. If $\mathscr {Q}^{k}_i$ are the control points of the polygon at *kth* level and $\mathscr {Q}^{k+1}_i$ are the control points at the $(K+1)$th level, then the 6-points interpolating binary subdivision scheme proposed by^25^ is defined as

4 $$\begin{aligned}{} & {} \mathscr {Q}_{2i}^{k+1}=\mathscr {Q}_{i}^k, \nonumber \\{} & {} \mathscr {Q}_{2i+1}^{k+1}=\omega (\mathscr {Q}_{i-2}^{k}+\mathscr {Q}_{i+3}^{k})-\left( \frac{1}{16}+3\omega \right) (\mathscr {Q}_{i-1}^{k}+\mathscr {Q}_{i+2}^{k}) +\left( \frac{9}{16}+2\omega \right) (\mathscr {Q}_{i}^{k}+\mathscr {Q}_{i+1}^{k}). \end{aligned}$$

This scheme produces $C^{2}$-continuous curve for the range $0<\omega \le \frac{3}{256}$, particularly we choose $\omega =\frac{3}{256}$^17^, support width $(-5, 5)$. Also its degree generation, reproduction and approximation order is five and six respectively. Fundamental solution of scheme (4) with basis function is given in (5) and (6) respectively.

5 $$\begin{aligned} \mathscr {D}(i)=\left\{ \begin{array}{l} 1, \quad \text{ for }\quad i=0,\\ 0, \quad \text{ for }\quad i\ne 0, \end{array}\right. \end{aligned}$$

and

6 $$\begin{aligned} \mathscr {D}(x)=\Sigma \dot{a}_\rho \mathscr {D}(2x-\rho ), \end{aligned}$$

where $\dot{a}_\rho$ is the mask of the scheme (4). The first two derivatives of (6) are given in (7) by using similar method as^17^.

7 $$\begin{aligned} \left\{ \begin{array}{l} \dot{\mathscr {D}}(0)=0,\qquad \dot{\mathscr {D}}(\pm 1)=\mp \frac{272}{365},\qquad \dot{\mathscr {D}}(\pm 2)=\mp \frac{53}{365},\quad \dot{\mathscr {D}}(\pm 3)=\mp \frac{16}{1095},\quad \dot{\mathscr {D}}(\pm 4)=\mp \frac{1}{2920},\\ \ddot{\mathscr {D}}(0)=-\frac{295}{28}{,} \qquad \ddot{\mathscr {D}}(\pm 1)=\frac{712}{105}, \qquad \ddot{\mathscr {D}}(\pm 2)=-\frac{184}{105}, \qquad \ddot{\mathscr {D}}(\pm 3)=\frac{8}{35},\qquad \ddot{\mathscr {D}}(\pm 4)=\frac{3}{280}. \end{array}\right. \end{aligned}$$

## Subdivision collocation method (SCM)

In this section, building upon the basic concepts of the subdivision scheme and its derivative calculations, we have developed a Subdivision Collocation Method (SCM) for solving the proposed problem. Additionally, this section includes the stability analysis and error estimations.

### Numerical approximation

This section includes the detailed derivation of the Subdivision Collocation Method (SCM) for the proposed problem:

Consider a partition of [0, *L*] that is equally divided by the knots $x_{i}$ into *n* subintervals $[x_{i}, x_{i+1}]$ where $i=0, 1, \ldots , N$ such that $0=x_{0}<x_{1}< \cdots< x_{N-1}<x_{N}=L$. Hence the approximate solution *u*(*x*, *t*) of (1) to exact solution *U*(*x*, *t*) based on the collocation approach can be expressed as

8 $$\begin{aligned} u(x,t)=\sum \limits ^{N+4}_{j=-4} \frac{\mathscr {P}_{2,i}(x)}{a_{m-j}} \mathscr {C}_{j} \qquad 0\le x\le L,\qquad where \qquad a_{m-j}=\left\{ \begin{array}{ll} 1, &{} \quad \text{ if }\quad m\ne j,\\ 0< a_{m-j}< 1, &{}\quad \text{ if }\quad m=j \end{array}\right. \end{aligned}$$

$\mathscr {P}_{2,i}(x)=\mathscr {D}(\frac{x-x_{i}}{h})$ with $h= \frac{L}{N}$ is the basis function of (4) and $\mathscr {C}_j(t)$ are the time dependent quantities which are to be evaluated. Now it is possible to simplify the approximation of the function $u^k_i$ at the coordinates $(x_i,t_k)$ across the finite interval $[x_i,x_{i+1}]$ by

9 $$\begin{aligned} u^{k}_{i}= \sum \limits ^{i+4}_{j=i-4}\frac{\mathscr {P}_{2,i}(x)}{a_{m-j}} \mathscr {C}_j, \qquad m=i=0,1,2, \ldots ,N. \end{aligned}$$

with $\mathscr {P}_{2,i}(x_j)=\mathscr {D}(\frac{x_{i}-x_{j}}{h})$ and $m=i=0,1,2,\ldots , N$. To obtain the approximation of solution of values of $\mathscr {P}_{2,i}(x_j)$ and its derivative at the knots are needed. Since the values vanishes at the other knots. The approximations of the problem (1) at the time level at $t_{j+1}+h$ can be considered by

10 $$\begin{aligned} (u_{t})^{k}_{i}+(u_{x})^{k}_{i}+(1-\theta )f^{k}_{i}+\theta f_{i}^{k+1}=0,\,\,\, \qquad i=0,1,\ldots ,N \end{aligned}$$

where

11 $$\begin{aligned} f^{k}_{i}= & {} -(u_{xx})^{k}_{i}, \qquad \,\,\, i=0,1,2,\ldots ,N \end{aligned}$$

12 $$\begin{aligned} (u_{t})^{k}_{i}= & {} \frac{u^{k+1}_{i}-u^{k}_{i}}{\bigtriangleup t},\qquad \,\,\, i=0,1,2,\ldots ,N \end{aligned}$$

successive time levels are *k* and $k+1$ where $k=0,1,2, \ldots$, we will rearrange equations from (10)–(12) and the results will be displayed below after discretizing the first order time derivatives by forward difference scheme.

13 $$\begin{aligned}{} & {} \frac{u^{k+1}_{i}-u^{k}_{i}}{\bigtriangleup t}+\frac{\beta }{h}\sum \limits ^{i+4}_{j=i-4}\mathscr {C}^{k}_{j}\frac{\mathscr {P}'_{2,i}(x_{j})}{a_{m-j}} -\frac{(1-\theta )\alpha }{h^2}\sum \limits ^{i+4}_{j=i-4}\mathscr {C}^{k}_{j}\frac{\mathscr {P}''_{2,i}(x_{j})}{a_{m-j}} -\frac{\theta \alpha }{h^2}\sum \limits ^{i+4}_{j=i-4}\mathscr {C}^{k+1}_{j}\frac{\mathscr {P}''_{2,i}(x)}{a_{m-j}}=0,\qquad \,\,\nonumber \\{} & {} \quad m= i=0,1,2,\ldots ,N \end{aligned}$$

where $\bigtriangleup t$ is the step size. Here Crank Nicloson approach is used to simplify the problem for $\theta =0.5$, hence Eq. (13) takes the form

14 $$\begin{aligned} \frac{u^{k+1}_{i}-u^{k}_{i}}{\bigtriangleup t}+\frac{\beta }{h}\sum \limits ^{i+4}_{j=i-4}\mathscr {C}^{k}_{j}\frac{\mathscr {P}'_{2,i}(x)}{a_{m-j}}-\left( \frac{\alpha }{2h^2}\right) \sum \limits ^{i+4}_{j=i-4}\mathscr {C}^{k}_{j}\frac{\mathscr {P}''_{2,i}(x)}{a_{m-j}}-\left( \frac{\alpha }{2h^2}\right) \sum \limits ^{i+4}_{j=i-4}\mathscr {C}^{k+1}_{j}\frac{\mathscr {P}''_{2,i}(x)}{a_{m-j}}=0, \end{aligned}$$

where $i=m=0,1,2,3, \ldots ,N$. This above calculation leads to the generation of a linear system of order $(N+1) \times (N+9)$ with $(N+9)$ unknowns at each level of time.

$$\begin{aligned} \mathscr {C}^{k+1}=(\mathscr {C}^{k+1}_{-4},\mathscr {C}^{k+1}_{-3},\mathscr {C}^{k+1}_{-2},\ldots ,\mathscr {C}^{k+1}_{N+4})^{T}. \end{aligned}$$

Using the notation $\mathscr {P}'_{2,i}(x_{j})=\dot{\mathscr {D}}(i-j)$ , $\mathscr {P}''_{2,i}(x_{j})=\ddot{\mathscr {D}}(i-j)$ , (14) becomes

$$\begin{aligned} u^{k+1}_{i}-u^{k}_{i}+\left( \frac{\beta \bigtriangleup t}{h}\right) \sum \limits ^{i+4}_{j=i-4}\mathscr {C}^{k}_{j}\frac{\dot{\mathscr {D}}(i-j)}{a_{m-j}}-\left( \frac{\alpha \bigtriangleup t}{2h^2}\right) \sum \limits ^{i+4}_{j=i-4}\mathscr {C}^{k}_{j}\frac{\ddot{\mathscr {D}}(i-j)}{a_{m-j}}-\left( \frac{\alpha \bigtriangleup t}{2h^2}\right) \sum \limits ^{i+4}_{j=i-4}\mathscr {C}^{k+1}_{j}\frac{\ddot{\mathscr {D}}(i-j)}{a_{m-j}}=0, \end{aligned}$$

this implies

15 $$\begin{aligned} u^{k+1}_{i}-\left( \frac{\alpha \bigtriangleup t}{2h^2}\right) \sum \limits ^{i+4}_{j=i-4}\mathscr {C}^{k+1}_{j}\frac{\ddot{\mathscr {D}}(i-j)}{a_{m-j}}=u^{k}_{i}-\left( \frac{\beta \bigtriangleup t}{h}\right) \sum \limits ^{i+4}_{j=i-4}\mathscr {C}^{k}_{j}\frac{\dot{\mathscr {D}}(i-j)}{a_{m-j}}+\left( \frac{\alpha \bigtriangleup t}{2h^2}\right) \sum \limits ^{i+4}_{j=i-4}\mathscr {C}^{k}_{j}\frac{\ddot{\mathscr {D}}(i-j)}{a_{m-j}}, \end{aligned}$$

now for $i=m=0$ in Eq. (15), we have

16 $$\begin{aligned} u^{k+1}_{0}-\left( \frac{\alpha \bigtriangleup t}{2h^2}\right) \sum \limits ^{+4}_{j=-4}\mathscr {C}^{k+1}_{j}\frac{\ddot{\mathscr {D}}(-j)}{a_{-j}}=u^{k}_{0}-\left( \frac{\beta \bigtriangleup t}{h}\right) \sum \limits ^{+4}_{j=-4}\mathscr {C}^{k}_{j}\frac{\dot{\mathscr {D}}(-j)}{a_{-j}}+\left( \frac{\alpha \bigtriangleup t}{2h^2}\right) \sum \limits ^{+4}_{j=-4}\mathscr {C}^{k}_{j}\frac{\ddot{\mathscr {D}}(-j)}{a_{-j}}, \end{aligned}$$

for $i=m=1$, Eq. (15), implies

17 $$\begin{aligned} u^{k+1}_{1}-\left( \frac{\alpha \bigtriangleup t}{2h^2}\right) \sum \limits ^{+5}_{j=-3}\mathscr {C}^{k+1}_{j}\frac{\ddot{\mathscr {D}}(1-j)}{a_{1-j}}=u^{k}_{1}-\left( \frac{\beta \bigtriangleup t}{h}\right) \sum \limits ^{+5}_{j=-3}\mathscr {C}^{k}_{j}\frac{\dot{\mathscr {D}}(1-j)}{a_{1-j}}+\left( \frac{\alpha \bigtriangleup t}{2h^2}\right) \sum \limits ^{+5}_{j=-3}\mathscr {C}^{k}_{j}\frac{\ddot{\mathscr {D}}(1-j)}{a_{1-j}}, \end{aligned}$$

similarly continuing the sequence for $i=m= N-1$, Eq. (15), becomes

18 $$\begin{aligned}{} & {} u^{k+1}_{N-1}-\left( \frac{\alpha \bigtriangleup t}{2h^2}\right) \sum \limits ^{N+3}_{j=N-5}\mathscr {C}^{k+1}_{j}\frac{\ddot{\mathscr {D}}(N-1-j)}{a_{N-1-j}}=u^{k}_{N-1}-\left( \frac{\beta \bigtriangleup t}{h}\right) \sum \limits ^{N+3}_{j=N-5}\mathscr {C}^{k}_{j}\frac{\dot{\mathscr {D}}(N-1-j)}{a_{N-1-j}}\nonumber \\{} & {} \quad +\left( \frac{\alpha \bigtriangleup t}{2h^2}\right) \sum \limits ^{N+3}_{j=N-5}\mathscr {C}^{k}_{j}\frac{\ddot{\mathscr {D}}(N-1-j)}{a_{N-1-j}}, \end{aligned}$$

for $i=m=N$, Eq. (15), implies

19 $$\begin{aligned}{} & {} u^{k+1}_{N}-\left( \frac{\alpha \bigtriangleup t}{2h^2}\right) \sum \limits ^{N+4}_{j=N-4}\mathscr {C}^{k+1}_{j}\frac{\ddot{\mathscr {D}}(N-j)}{a_{N-j}}=u^{k}_{0}-\left( \frac{\beta \bigtriangleup t}{h}\right) \sum \limits ^{N+4}_{j=N-4}\mathscr {C}^{k}_{j}\frac{\dot{\mathscr {D}}(N-j)}{a_{N-j}}\nonumber \\{} & {} \quad +\left( \frac{\alpha \bigtriangleup t}{2h^2}\right) \sum \limits ^{N+4}_{j=N-4}\mathscr {C}^{k}_{j}\frac{\ddot{\mathscr {D}}(N-j)}{a_{N-j}}, \end{aligned}$$

from the above equations we got the system of equation and matrix form

20 $$\begin{aligned} W^{k+1}-\left( \frac{\alpha \bigtriangleup t}{2h^2}\right) A\mathscr {C}^{k+1}=W^{k}-\left( \frac{\beta \bigtriangleup t}{h}\right) B\mathscr {C}^{k}+\left( \frac{\alpha \bigtriangleup t}{2h^2}\right) G\mathscr {C}^{k}, \end{aligned}$$

where $$W^{k+1}=(u^{k+1}_{0},u^{k+1}_{1},u^{k+1}_{2},\ldots ,u^{k+1}_{N})^{T}$$ and    $$W^{k}=(u^{k}_{0},u^{k}_{1},u^{k}_{2},\ldots ,u^{k}_{N})^{T}.$$

After some simplification of $W^{k+1}$ and $W^{k}$ with the help of (8), Eq. (20) can be transformed as

21 $$\begin{aligned} -A\mathscr {C}^{k+1}=F\mathscr {C}^{k} \qquad where \qquad F=\left( -\frac{\beta \bigtriangleup t}{h}\right) B+\left( \frac{\alpha \bigtriangleup t}{2h^2}\right) G, \end{aligned}$$

Eq. (21), implies

22 $$\begin{aligned} \mathscr {C}^{k+1}= & {} E\mathscr {C}^{k} \qquad where \qquad E=-A^{-1} F \nonumber \\ \mathscr {C}^{k+1}= & {} (\mathscr {C}^{k+1}_{-4},\mathscr {C}^{k+1}_{-3},\mathscr {C}^{k+1}_{-2},\ldots ,\mathscr {C}^{k+1}_{N+4})^{T} \quad \text {and} \quad \mathscr {C}^{k}=(\mathscr {C}^{k}_{-4},\mathscr {C}^{k}_{-3},\mathscr {C}^{k}_{-2},\ldots ,\mathscr {C}^{k}_{N+4})^{T} \end{aligned}$$

for $$k=1,2,3,\ldots , N+1$$       and       $$b=1,2,3,\ldots ,N+9$$

23 $$\begin{aligned} A=[\gamma ^{(\mu b)}_{b-\mu -4}]_{(N+1)\times (N+9)} \qquad \text{ and } \qquad \qquad \qquad F=[\varphi ^{(\mu b)}_{b-\mu -4}]_{N+1\times N+9} \end{aligned}$$

with

$$\begin{aligned} {\gamma }(l)=\left\{ \begin{array}{ll} \ddot{\mathscr {D}}_{l}, &{}\quad \text{ if }\quad l\in [-4,0)\quad \text{ and }\quad (0,4]\\ 0, &{}\quad \text{ if }\quad l\notin [-4,4]\\ \frac{\ddot{\mathscr {D}}_{l}}{a_{0}}-\frac{2h^2}{\alpha \bigtriangleup t}, &{}\quad \text{ if }\quad l=0 \end{array}\right. ,\qquad {\varphi }(l)=\left\{ \begin{array}{ll} \ddot{\mathscr {D}}_{l}+\dot{\mathscr {D}}_{l}, &{} \quad \text{ if }\quad l\in [-4,0)\quad \text{ and }\quad (0,4]\\ 0, &{}\quad \text{ if }\quad l\notin [-4,4]\\ \frac{\ddot{\mathscr {D}}_{l}}{a_{0}}+\frac{2h^2}{\alpha \bigtriangleup t}, &{}\quad \text{ if }\quad l=0 \end{array}\right. \end{aligned}$$

### Formation of consistent system

Since the order of the coefficient matrix of system (22) is $(N+1)\times (N+9)$. After adding the given boundary conditions to the system (22), the order of the coefficient matrix increases to $(N+3)\times (N+9)$. However, the system remains inconsistent. To attain a unique solution, six additional conditions are required. These conditions are constructed as follows:

Since the degree of reproduction of subdivision scheme (4) is five, so we have established six-order boundary conditions to ensure a unique solution. For simplicity, we will only discuss the left end points, namely $c_{-3}, c_{-2}, c_{-1}$. The right end points $c_{N+1}, c_{N+2}, c_{N+3}$ are handled in a similar manner.

The values $c_{-3}, c_{-2}, c_{-1}$ are determined by the quintic polynomial interpolating the points$(u_r, c_r)$, $0\le r\le 5$. i.e. The conditions at the left end are derived from

$$\begin{aligned} c_{-r}=M(-u_r), \quad r=1,2,3, \end{aligned}$$

where

24 $$\begin{aligned} M(u_r)=\sum \limits C(6,q)(-1)^{q+1}\mathbb {C}(u_{r-q}).\quad q=1,2,3,4,5,6 \end{aligned}$$

Since by (8), $\mathbb {C}(u_r)=c_r$ for $r=1,2,3$ and substituting $u_r$ by $-u_r$ in (24), we have

$$\begin{aligned} M(-u_r)=\sum \limits C(6,q)(-1)^{q+1}c_{r-q}.\quad q=1,2,3,4,5,6 \end{aligned}$$

By the equation given below we can get three left end conditions

25 $$\begin{aligned} \sum \limits C(6,q)(-1)^{q}c_{q-r}=0, r=3,2,1 \quad q=1,2,3,4,5,6 \end{aligned}$$

Using same pattern as left end conditions, we have following three right end conditions

26 $$\begin{aligned} \sum \limits C(6,q)(-1)^{q}c_{r-q}=0,\,\ r=N+3,N+2,N+1\quad q=1,2,3,4,5,6 \end{aligned}$$

After obtaining these left and right end conditions the system of nonlinear equations of order $(N+9)\times (N+9)$ will be formed

27 $$\begin{aligned} C^{k+1}=\mathscr {J}_{c}C^{k}, \end{aligned}$$

where

28 $$\begin{aligned} \mathscr {J}_{c}=(\mathscr {L}_{c_{0}}^T, {E}^T, \mathscr {R}_{c_{N}}^T). \end{aligned}$$

where *E* is represented in (22), The first three rows of the matrix $\mathscr {L}_{ c_ {0}}$ are derived from all the requirements stated at the domain’s left end from (25). And the fourth row of $\mathscr {L}_{c_{0}}$ is formed from (3) at $\mathscr {C}(0)=u_{0}=\phi _{1}(t)$. Similarly the last three rows of the matrix $\mathscr {R}_{c_{N}}$ are formed from all of the requirements that have been obtained at the right end of the domain from the (26). First row comes from (3) at $\mathscr {C}(N)=u_{L}=\phi _{2}(t)$. Hence The column vector $\mathscr {M}$ is defined as

29 $$\begin{aligned} \mathscr {M}=(0, 0, 0, \mathscr {C}(0), ({C}^{k+1})^{T}, \mathscr {C}(N), 0, 0, 0)^{T} \end{aligned}$$

where $C^{k+1}$ is given in (22).

### Initial guess

Numerous iterative algorithms heavily depend on the initial guess, and it has a profound impact on the stability, speed, and reliability of the algorithm. When employing numerical and computational methods, the process of selecting a suitable initial guess is often intricate and problem-specific, and its significance cannot be underestimated. Selecting the appropriate initial guess, suited to the particular requirements of each scenario, is unquestionably a crucial step in tackling a wide range of mathematical and computational issues.

To initiate the iterative process (27), we develop an efficient method for computing the initial guess, which supports the convergence of iterative algorithms. The initial guess, denoted as $C^{(0)} = [C_{-4}, C_{-3}, \ldots , C_{N+3}, C_{N+4}]^T$, is constructed from the initial and boundary conditions of the given problem as follows:

At the initial time level, the approximate solution, as derived from (8), takes the form,

30 $$\begin{aligned} u(x,0)=\sum \limits ^{N+4}_{s=-4}C_{s}\mathscr {P}_{2,s} \qquad 0\le x\le l, \end{aligned}$$

where $C_{s}'s$ are unknown. For the determination of $C_{s}$, the following conditions must be satisfied to ensure the correctness the initial approximation (30) as

$$\begin{aligned} u(x_s,0)=\psi _{1}(x_s),\qquad \qquad u(x_0,0)=\phi _{1}(t),\qquad u(x_N,0)=\phi _{2}(t),\qquad s=0,1, \ldots ,N \end{aligned}$$

where $x_{s}=sh$. As a result $(N+9)\times (N+9)$ matrix system

31 $$\begin{aligned} \mathscr {J}_{c}C^{0}=H, \,\ \text{ with } \,\ H=[0,0,0,\phi _{1}(t),\psi _{1}(x_0),\ldots , \psi _{1}(x_N),\phi _{2}(t),0,0,0]^T_{N+9} \end{aligned}$$

where $\psi _{1}(x)$, $\phi _{1}(t) \,\ \text {and} \,\ \phi _{2}(t)$ and $\mathscr {J}_{c}$ are given in (2), (3) and (28) respectively. Hence, the initial guess for the given problem will be obtained by solving Eq. (31).

### Iterative algorithm

The iterative algorithm is defined in detail as follows:

Step 1 **Initial Solution**:
  Calculate the initial solution, $C^{0}$, by solving the system (31) using any numerical method.
Step 2 **Iterative Algorithm: **
  32 $$\begin{aligned} C^{k+1}=\mathscr {J}_{c} C^k, \qquad k=0,1,2,3, \ldots , \end{aligned}$$
Step 3 **Stopping/Convergenc Criteria**:
  If $\varepsilon =10^{-6}$ is the given error tolerance, the iterative process will terminate when the following condition is met.
  33 $$\begin{aligned} \Vert C^k-C^{k-1}\Vert \le \varepsilon , \qquad k=0,1,2,3, \ldots , \end{aligned}$$

**Figure 1:**
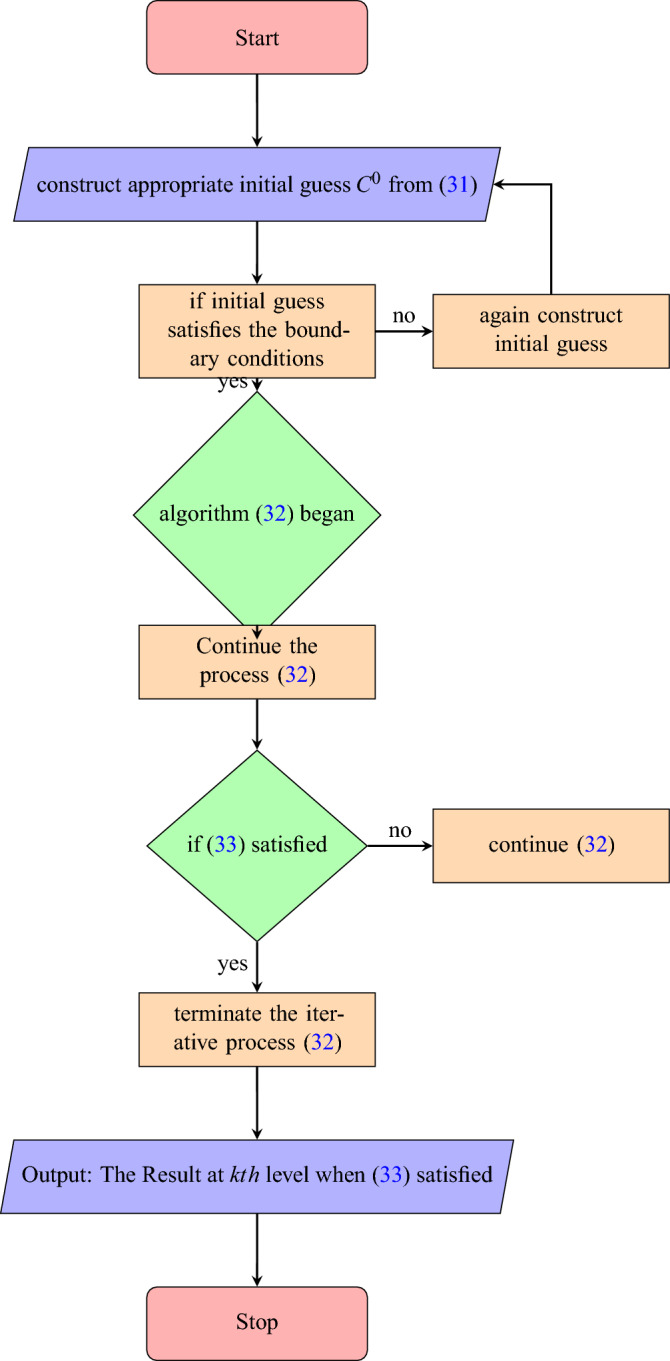
Flowchart of iterative algorithm.

The iterative algorithm is defined in the form of a flowchart given Fig. 1.

## Stability analysis and estimation of errors

### Stability analysis

#### Theorem 1

*The iterative algorithm* (32) *converges for* $\frac{4h^2}{\alpha \bigtriangleup t}>0$ *or* $\alpha >0$ *and* $Z>0$.

#### Proof

The Von-Neumann stability approach provide us growth of error in as single Fourier mode. For this we use,

34 $$\begin{aligned} C^{n}_{\nu }=\exp (\imath \eta \nu h), \end{aligned}$$

where $\imath =\sqrt{-1}$ and $\eta$ is a real number. Von Neumann stability analysis is used to analyze the convergence of linearize scheme.

We consider

35 $$\begin{aligned} \sum \limits ^{4}_{\nu =-4}\ddot{\mathscr {D}}({\nu })C^{k+1}_{\nu }=\sum \limits ^{4}_{\nu =-4}(\ddot{\mathscr {D}}+\dot{\mathscr {D}})({\nu })^{'}C^{k}_{\nu }. \end{aligned}$$

Substitution of (34) in (35) implies

$$\begin{aligned} \sum \limits ^{4}_{\nu =-4}\ddot{\mathscr {D}}({\nu })\exp (i\eta \nu h)=\sum \limits ^{4}_{\nu =-4}(\ddot{\mathscr {D}}+\dot{\mathscr {D}})({\nu })^{'}\exp (i\eta \nu h). \end{aligned}$$

Since

$$\begin{aligned} \xi =\frac{F+iG}{F'+iG'}, \end{aligned}$$

this implies

36 $$\begin{aligned} |\xi |=\left| \frac{F+iG}{F'+iG'}\right| \le 1, \end{aligned}$$

where

$$\begin{aligned} F=\frac{3}{280}cos4\eta h+\frac{8}{35}cos3\eta h-\frac{184}{105}cos2\eta h+\frac{712}{105}cos\eta h-\frac{295}{28a_{0}}-\frac{2h^2}{\alpha \bigtriangleup t},\qquad G=0, \end{aligned}$$

and

$$\begin{aligned} F'=\frac{3}{280}cos4\eta h+\frac{8}{35}cos3\eta h-\frac{184}{105}cos2\eta h+\frac{712}{105}cos\eta h-\frac{295}{28a_{0}}+\frac{2h^2}{\alpha \bigtriangleup t}, \qquad G'=0. \end{aligned}$$

The above equations can be written as

37 $$\begin{aligned} F=Z-\frac{2h^2}{\alpha \bigtriangleup t},\qquad G=0, \quad \text{ and } \quad F'=Z+\frac{2h^2}{\alpha \bigtriangleup t}, \qquad G'=0, \end{aligned}$$

where

38 $$\begin{aligned} Z= \frac{3}{280}cos4\eta h+\frac{8}{35}cos3\eta h-\frac{184}{105}cos2\eta h+\frac{712}{105}cos\eta h-\frac{295}{28a_{0}} \end{aligned}$$

Using (37) and (38) in (36), we get

39 $$\begin{aligned} \left| \frac{ Z-\frac{2h^2}{\alpha \bigtriangleup t}}{Z+\frac{2h^2}{\alpha \bigtriangleup t}}\right| <1, \end{aligned}$$

From (39), it is concluded that the system is convergent for $\frac{4h^2}{\alpha \bigtriangleup t}>0$ or $\alpha >0$ and $Z>0$ the method. $\square$

### Error estimation

The errors between the exact solutions $U_{i}$ and the numerical solutions $u_{i}$ are estimated at $x_{i}$ by calculating the absolute error , average error , $L_2$, root mean square error, maximum relative error are defined as follows:

The absolute error is denoted by $Error _{ABS}$ and estimated as

40 $$\begin{aligned} Error _{ABS}=\parallel U_{i}-u_{i}\parallel _{\infty }. \end{aligned}$$

The average error is denoted by $Error _{AVG}$ and is estimated as

41 $$\begin{aligned} Error_{AVG}= & {} \frac{\sum \nolimits _{i=1}^{N}\parallel U_{i}-u_{i}\parallel _{\infty }}{N}, \end{aligned}$$

42 $$\begin{aligned} L _{2}= & {} \parallel U_{i}-u_{i}\parallel _{2}. \end{aligned}$$

The root mean square error is denoted by *RMSE* and is estimated as

43 $$\begin{aligned} RMSE= \sqrt{\frac{\sum \nolimits _{i=1}^{N}\parallel U_{i}-u_{i}\parallel _{\infty }}{N}}, \end{aligned}$$

where *N* represents the total number of node points. The maximum relative error is denoted by *MRE* and is estimated as

44 $$\begin{aligned} MRE=\left\| \frac{U_{i}-u_{i}}{U_{i}}\right\| _{\infty } \end{aligned}$$.

### Computational order

Computational order (C-order) of the subdivision collocation method presented with the following formula

$$\begin{aligned} \text{C-order}=\frac{\log \left| \frac{E_{i+1}}{E_{i}}\right| }{\log \left| \frac{E_i}{E_{i-1}}\right| }, \end{aligned}$$

where $E_{i-1}$, $E_{i}$ and $E_{i+1}$ are the errors at consecutive iterations, for grid size *h*.

## Numerical examples, results and discussion

The numerical approach suggested in the preceding part is demonstrated here. In this section using the subdivision collocation algorithm to find solution of the different problems. Computations are carried out in Matlab. The numerical solutions for each example are calculated as follows: we first computed an initial guess by solving (31), and then, using the obtained initial guess, the numerical solution are obtained through an iterative algorithm (32)–(33) for $\alpha >0$, $h > 0$ and $\bigtriangleup t>0$. In Examples 1–5, if the advection time scale is less than the diffusion time scale the transport is advection controlled this corresponds $P_{e}>>1$, and if $P_{e}<<1$ then diffusion dominates.

### Numerical examples

#### Example 1

We consider transport equation (1) with flow velocity $\beta =2$ and diffusion coefficient $|\alpha |=|\beta |/P_{e}$, where $P_{e}=P\grave{e}$clet number, in a channel length $L=2$ of constant cross section and a bottom slop with physical conditions^1^ initial condition: $\psi _{1}(x)=exp({\frac{(x-\beta )^2}{4\alpha }})$ boundary conditions: $\phi _{1}(t)=\frac{1}{\sqrt{(1+t)}} exp{\frac{(-(1+t)\beta )^2}{4\alpha (1+t)}}$ and $\phi _{2}(t)=\frac{1}{\sqrt{(1+t)}} exp{\frac{(L-(1+t)\beta )^2}{4\alpha (1+t)}}$. The exact solution is $\frac{1}{\sqrt{(1+t)}} exp{\frac{(x-(1+t)\beta )^2}{4\alpha (1+t)}}$.

#### Example 2

We consider transport equation (1) taken from^1,23,24^ with different values of flow velocity $\beta$, diffusion coefficient in channel length *L* of constant cross section and physical conditions, initial condition: $\psi _{1}(x)=a e^{(-\bar{c}x)}$ and boundary conditions: $\phi _{1}(t)=a e^{(bt)}$ and $\phi _{2}(t)= a e^{(bt-\bar{c}L)}$. The exact solution is given by $a e^{(bt-\bar{c}x)}$, where $a=1$, $b=0.1, 0.2$ and $\bar{c}=\frac{-\beta +\sqrt{\beta ^2+4\alpha b}}{2\alpha }$.

#### Example 3

We consider transport equation (1) taken from^1,2,6,23^, with different values of flow velocity $\beta$, diffusion coefficient in channel length *L* of constant cross section and physical conditions, initial condition: $\psi _{1}(x)=sinx,$ and two types of boundary conditions,

Type I: $\phi _{1}(t)=e^{(-\alpha t)}sin(-\beta t)$ and $\phi _{2}(t)=e^{(-\alpha t)}sin(L-\beta t)$ known as Dirichlet boundary conditions.
Type II: $\phi '_{1}(t)=e^{(-\alpha t)}cos(\beta t)$ and $\phi '_{2}(t)=e^{(-\alpha t)}cos(L-\beta t)$ known as Neumann boundary conditions taken from Example 6 of^2^. The exact solution is $e^{(-\alpha t)}sin(x-\beta t)$.

#### Example 4

We consider transport equation (1) taken from^1–5^ with initial condition $\psi _{1}(x)=exp(\frac{5x}{2})(cos(\pi x/2)+0.25sin(\pi x/2)),$ $\phi _{1}(t)=exp\left( \frac{-5t}{2}-\frac{\pi ^2t}{40}\right)$ and $\phi _{2}(t)=exp\left( \frac{5(L-t)}{2}\right) exp\left( -\frac{\pi ^2t}{40}\right) \left( cos(\pi L/2)+0.25sin(\pi L/2)\right) )$ are the boundary conditions. The exact solution is $exp\left( \frac{5(x-t)}{2}\right) exp\left( -\frac{\pi ^2t}{40}\right) \left( cos(\pi x/2)+0.25sin(\pi x/2)\right. )$.

#### Example 5

We consider transport equation (1) taken from^7,8^ having $L=9$ with constant cross section and bottom slope with the physical condition, $\psi _{1}(x)=exp(-(x-1)^2/\alpha ),$ and boundary conditions given below, $\phi _{1}(t)=1/\sqrt{4t+1}\,\, exp{[-(-1-\beta t)^2/\alpha (4t+1)]}\qquad \text{ and }\qquad \phi _{2}(t)=1/\sqrt{4t+1}\,\,exp{[-(L-1-\beta t)^2/\alpha (4t+1)]},$ and the exact solution is $1/\sqrt{4t+1}\,\,exp{[-(x-1-\beta t)^2/\alpha (4t+1)]}$.

### Numerical and graphical findings

**Figure 2:**
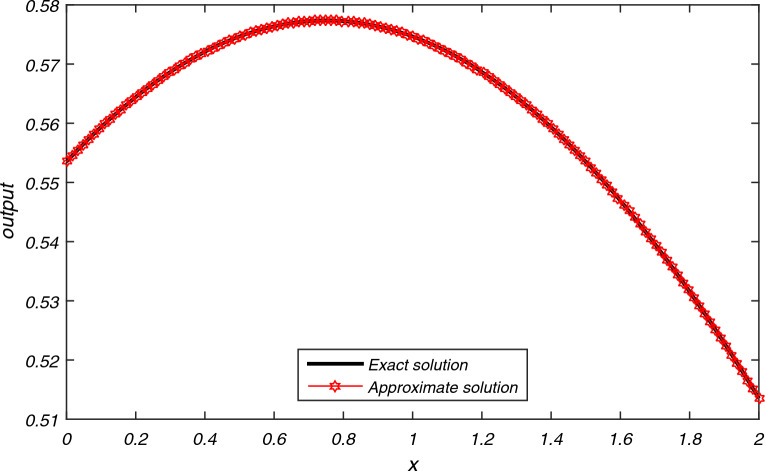
Approximate solution of problem 1 at $P_{e}=2.5$ and $T=1$, $h=1/128$.

**Figure 3:**
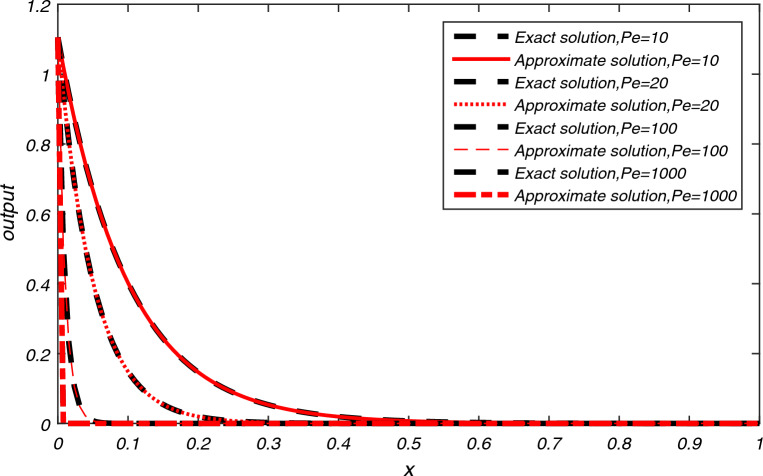
Approximate solution of Example 2 at $P_{e}=1000$, $T=1, h=1/64$.

**Figure 4:**
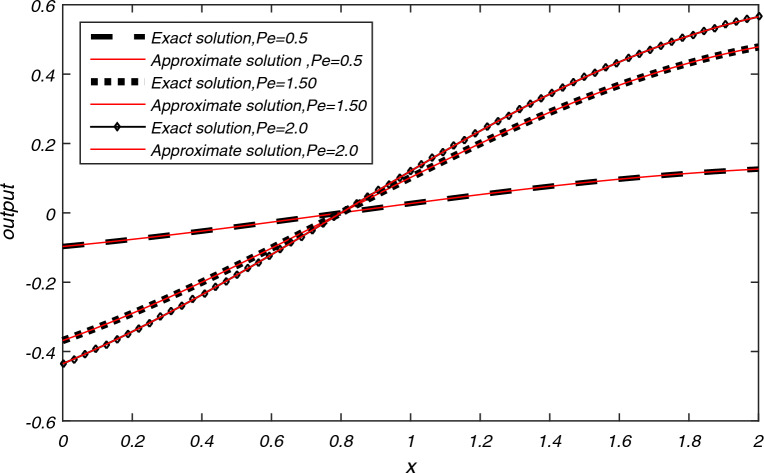
Exact and approximate solutions of Example 3 at different $P_{e}$ numbers.

**Figure 5:**
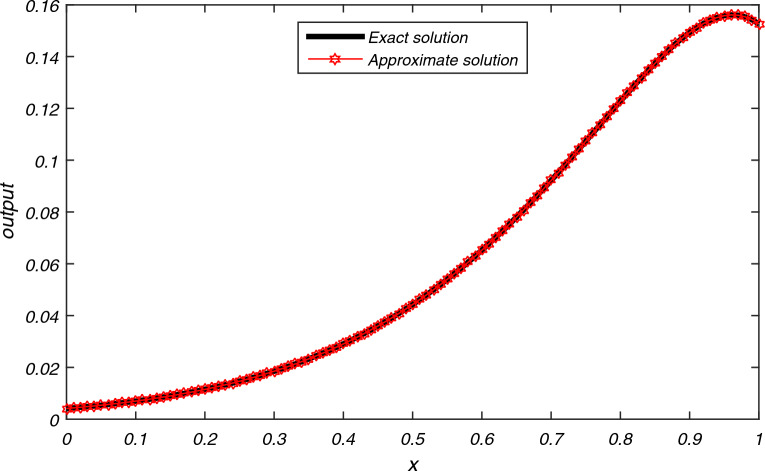
Exact and approximate solutions of Example 4 for $h=0.01, \bigtriangleup t=2h, T=2, L=1$.

**Figure 6:**
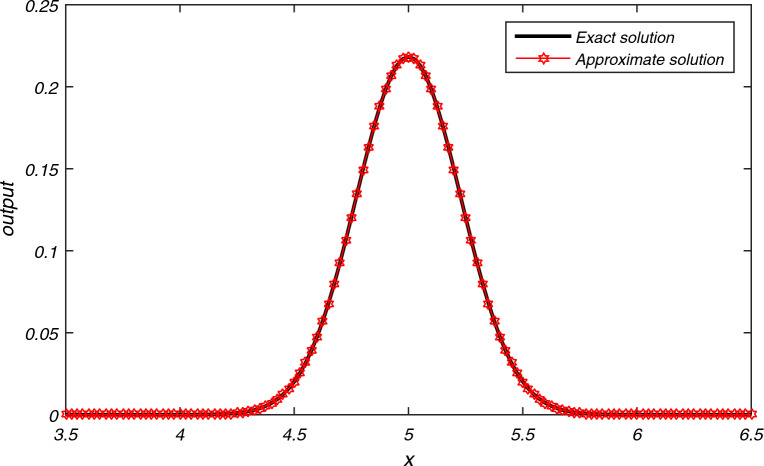
Exact and approximate solutions of problem 5 for $h=0.025, \bigtriangleup t=0.005, t=5$.

- The computational results of Example 1 are presented in Tables 1, 2, 3 for flow velocity $\beta =0.25$, $\Delta t=2h$, $L=2$ and $N=200$. Table 1 displays the comparison between the exact and approximate solutions for $h=0.01$, $t=2$, and $P_{e}=2.5$, while also illustrating the accuracy and efficiency of the present algorithm through the absolute errors and CPU time = 0.337 s. Tables 2 and 3 display average error norms, computational order and CPU time for various choices of *h* with $P_e$ values of 2.5, 25, 250, and 2500 using the present method. They are compared with average errors reported in^1^. Both tables reveal that the present method yields good results for each value of $P_e$. Figure 2 presents the exact and approximate solutions plot for $P_{e} = 2.5$, $T = 1$, and $h = \frac{1}{128}$. From Fig. 2, we can observe that the numerical results of the present method closely align with the exact solutions.
- The computational results for Example 2 are presented in Tables 4, 5, 6 and 7 for various values of flow velocity $\beta$ diffusion coefficient $\alpha$, length *L* and $P_{e}$. Table 4 displays the comparison between the exact and approximate solutions for $\beta =-1$, $L=1$, $h=0.01$, $t=1$, and $P_{e}=10$, while also illustrating the accuracy and efficiency of the present algorithm through the absolute errors and CPU time = 0.393 s for $N=200$. The Table 5 display the comparison between the exact and approximate solutions as well as comparison with existing methods^23,24^. The average errors for Example 2 obtained using the present method with parameters $a=1$, $b=0.1$, flow velocity $\beta = -1$, time step $\Delta t = 2h$, $L = 1$, and $T = 1$ are compared to existing methods^1^ for various values of $P_{e}$ are presented in Tables 6 and 7. The data presented in the tables clearly indicates that as values of $P_{e}$ increases, advection gains more control, resulting in smoother transport compared to the methods in^1,23,24^. A graph depicting the exact and approximate solutions for Example 2 at different values of $P_e$ is shown in Fig. 3. This comparison unequivocally establishes that the present method consistently provides significantly more accurate results.
- The computational results for Example 3, with Dirichlet boundary conditions, are presented in Table 8. The results for flow velocity $\beta =1$, a diffusion coefficient of $\alpha =1/1000$, a length of $L=2$, a spatial step size of $h=0.01$, a time interval of $t=1$, a time step size of $\Delta t=0.05h$, and $Pe=1000$. The table compares the exact and approximate solutions, illustrating the accuracy and efficiency of the present algorithm through absolute errors. The CPU time for $N=100$ is recorded at 0.393 s.
  The computation of absolute error results for Example 3 with Dirichlet boundary conditions, obtained using the present SCM, with the following parameters: flow velocity $\beta =1$, time step $\Delta t=0.05h$, length $L=2$, and $T=1$, are compared to existing methods^2,6,23^ for various values of $P_{e}$ are presented in Tables 9, 10. Figure 4 represents the exact and approximate solution plots for various values of $P_{e}=0.5, 1.50, 2.0$, with $h=1/64$, and $T=1$.
  The computation of absolute error $L_{\infty }$ and $L_{2}$ for Example 3 with Neumann boundary conditions are presented in Table 11. These results are obtained for a flow velocity of $\beta =0.1$, time step $\Delta t=0.1h$, and a length of $L=2$. They are compared to the methods in^2,6^ for different values of *T*. It is evident that the computational data and graphical representation of the current method closely align with the exact solution for different values of $P_{e}$.
  In both Dirichlet and Neumann boundary value problems, the transport is advection-controlled, rapid, and smooth, except in Table 9 where, for $P_e=0.5<1$, the diffusion term dominates.
- The computational results of Example 4 are presented in Tables 12, 13, 14 for $\beta =1$ and $L=1 \,\ \text {and} \,\ 2$. Table 12 displays the comparison between the exact and approximate solutions for $h=0.01$, $t=2$, $L=1$, $N=100$ and $P_{e}=10$, while also illustrating the accuracy and efficiency of the present algorithm through the absolute errors and CPU time = 0.339 s. The computation of absolute error results for Example 4 using the present method and a comparison of absolute errors with existing methods^1–5^ are presented in Tables 13 and 14. These results are obtained for a diffusion coefficient of $\alpha =0.1$, a flow velocity of $\beta =1$, a time step of $\Delta t=2h$, a length of $L=1$, and a time duration of $T=2$, for different values of $h=0.1, 0.05, 0.01$. A graphical representation of the exact and approximate solutions for $h=0.01, L=1, T=2$ is depicted in Fig. 5.
  The numerical data and graphical representation closely match the exact solution. In Example 4, transportation is advection-controlled and more efficient than existing methods.
- The numerical solution for Example 5, obtained within a CPU time of 0.389 seconds using the present method with values of flow velocity $\beta =0.8$, diffusion coefficient $\alpha =0.005$, $\Delta t=0.005$, $h=0.025$, $T=5$, and $N=100$, is presented in Table 15, and it is compared to existing methods^7,8^. The graph in Fig. 6 illustrates the exact and approximate solutions for Example 5 with $h=0.025$ and $T=5$. Given these parameters, it is evident that the advection time scale is shorter than the diffusion time scale, indicating that transportation is advection-controlled and very fast.

**Table 1:** Comparison between the exact and approximate solutions of Example 1 using the Present SCM for $P_{e}=2.5, h=0.01, t=2, \bigtriangleup t=2h, L=2$.

Grid points	Exact solutions	Approximate solution
0.00	$5.53 \times {10^{-1}}$	$5.53 \times {10^{-1}}$
0.25	$5.67\times {10^{-1}}$	$5.67\times {10^{-1}}$
0.50	$5.75\times {10^{-1}}$	$5.75\times {10^{-1}}$
0.75	$5.77\times {10^{-1}}$	$5.77\times {10^{-1}}$
1.00	$5.75\times {10^{-1}}$	$5.75\times {10^{-1}}$
1.25	$5.67\times {10^{-1}}$	$5.67\times {10^{-1}}$
1.50	$5.53\times {10^{-1}}$	$5.53\times {10^{-1}}$
1.75	$5.36\times {10^{-1}}$	$5.36\times {10^{-1}}$
2.00	$5.14\times {10^{-1}}$	$5.14\times {10^{-1}}$

**Table 2:** Comparison between the average errors of existing methods and the Present SCM of Example 1 at $P_{e}=2.5 \,\ \text {and} \,\ 25$.

*h*	for $P_{e}=2.5$				for $P_{e}=25$			
	$Error_{AVG}$ by SCM	By^1^	*C*-order by SCM	CPU time by SCM (s)	$Error_{AVG}$ by SCM	By^1^	*C*-order of SCM	CPU time by SCM (s)
1/16	$4.8072\times 10^{-10}$	$6.7927\times 10^{-7}$	9.31	0.302	$2.1921\times 10^{-9}$	$2.1239\times 10^{-4}$	8.62	0.249
1/32	$2.7514\times 10^{-10}$	$4.0770\times 10^{-8}$	7.65	0.270	$1.1874\times 10^{-9}$	$1.3249\times 10^{-5}$	7.14	0.262
1/64	$5.8214\times 10^{-10}$	$2.5470\times 10^{-9}$	6.06	0.318	$9.6835\times 10^{-10}$	$8.3479\times 10^{-7}$	5.92	0.269
1/128	$7.5145\times 10^{-11}$	$1.8370\times 10^{-10 }$	5.59	0.318	$3.6480\times 10^{-10}$	$5.3779\times 10^{-8}$	5.22	0.269

**Table 3:** Comparison between the average errors of existing methods and the Present SCM of Example 1 at $P_{e}=250 \,\ \text {and} \,\ 2500$.

*h*	For $P_{e}=250$				For $P_{e}=2500$			
	SCM	^1^	*C*-order by SCM	CPU time by SCM	SCM	^1^	*C*-order by SCM	CPU time by SCM
1/16	$2.5309\times 10^{-8}$	$1.8359\times 10^{-2}$	7.57	0.328s	$2.5649\times 10^{-7}$	$7.7555\times 10^{-2}$	6.57	0.241s
1/32	$1.3082\times 10^{-8}$	$2.9361\times 10^{-3}$	5.37	0.260s	$1.3253\times 10^{-7}$	$2.8025\times 10^{-2}$	5.50	0.251s
1/64	$6.6480\times 10^{-9}$	$2.2609\times 10^{-4}$	5.37	0.278s	$6.7334\times 10^{-8}$	$1.0947\times 10^{-2}$	4.72	0.242s
1/128	$3.3508\times 10^{-9}$	$1.4294\times 10^{-5}$	4.69	0.306s	$3.3935\times 10^{-8}$	$2.4936\times 10^{-3}$	4.14	0.263s

### Discussion on findings

In this research work, we took five examples from the literature and solve with current method and compare them with previously repointed methods^1–8,23,24^. Our technique demonstrates superior performance compared to other existing methods. In all examples, both computational and graphical results indicate that substances move rapidly rather than dispersing within the fluid. Additionally, it is observed that our current technique aligns well with the analytical solution compared to other methods.

- In Example 1, we compared our results with the numerical findings obtained by^1^. It is evident from the results presented in Tables 1, 2, 3 and 16 that the substances moved more swiftly compared to the results obtained by^1^, considering the constant flow velocity and diffusion coefficient. The substance concentrations are advected along the flow direction for the given parametric values, performing notably well within the specified interval $0<\beta \le 0.25$ for flow velocity and times $0<t \le 2$. Employing our present method with smaller values of *h* allows for an increase in the constant flow velocity’s magnitude, enabling swifter movement of substances within an extended channel.
- Numerical results obtained from Example 2 are compared with those obtained from^1,23,24^, presented in Tables 4, 5, 6, 7 and 16. The computational data indicates a consistent fluid flow due to the swift movement of substances within the specified channel length, as evidenced in the results obtained from other existing methods. The current technique excels in addressing the problem within a time range of $0 < t \le 1$ and a flow velocity range of $-1 < \beta \le 1$, utilizing the same parameters as those employed in Example 2. By employing smaller *h* values and extending the channel length with a higher constant flow velocity, this research method achieves uniform flow and expedites the removal of substances present in the fluid within a minimal timeframe.
- Tables 8, 9, 10 and 11 display the computational data of Example 3 along with the results obtained by the methods^2,6,23^. Table 16 represents the different types of errors obtained by present method. All these results demonstrate the accuracy of the present method, confirming a uniform flow of the fluid within the range of $0 < t \le 1$ and $0 < \beta \le 1$ for smaller values of *h*. Our research method yields favorable results within a short time period with a minimal velocity range for the same channel length. Moreover, it significantly improves the efficiency of moving substances within the fluid more rapidly and efficiently over longer distances with a high constant velocity. The method’s efficiency increases notably with smaller values of *h*.
- The numerical results of Example 4 are compared with those from^1–5^, presented in Tables 12, 13, 14. For the sake of comparison, the numerical results are obtained using the same grid points as those used in the existing methods. These results validate the swift transportation of substances and ensure a uniform flow of fluid within the specified channel length. Table 16 further validates the method’s accuracy through various error evaluations for $N=100$. The method’s accuracy remains consistent within the minimum ranges of $0<t\le 2$ and $0<\beta \le 1$, remaining unaffected by variations. Moreover, this research method demonstrates improved performance for longer distances when employing increased constant flow velocity and smaller values of *h*.
- The numerical results of Example 5, presented in Table 14, illustrate the uniform flow of the fluid based on the employed parameters. Additionally, Table 15 confirms the rapid movement of substances within the fluid compared to existing methods^7,8^. The accuracy of the current method for $N=100$ is presented in Table 16, showcasing various error computations. The computational data demonstrates the efficient performance of the present method, confirming the rapid transport of substances within the limited ranges of $0<t\le 5$ and $0<\beta \le 0.8$. Furthermore, increasing the channel length with smaller values of *h* and higher velocity does not significantly affect the accuracy of the present research method.
- Figures 2, 3, 4, 5 and 6 shows the uniform flow of fluid with less concentration of substances which does not effect flow of fluid.

**Table 4:** Comparison between the exact and approximate solutions of Example 2 using the Present SCM for $P_{e}=10, h=0.01, t=1, \bigtriangleup t=2h, L=1$.

Grid points	Exact solution	Approximate solution
0.0	$1.11\times 10^{0}$	$1.11\times 10^{0}$
0.1	$4.03 \times 10^{-1}$	$4.03\times 10^{-1}$
0.2	$1.47\times 10^{-1}$	$1.47\times 10^{-1}$
0.3	$5.34\times 10^{-2}$	$5.34\times 10^{-2}$
0.4	$1.95\times 10^{-2}$	$1.95\times 10^{-2}$
0.5	$7.09\times 10^{-3}$	$7.09\times 10^{-3}$
0.6	$2.58\times 10^{-3}$	$2.58\times 10^{-3}$
0.7	$9.40\times 10^{-4}$	$9.40\times 10^{-4}$
0.8	$3.43\times 10^{-4}$	$3.43\times 10^{-4}$
0.9	$1.25\times 10^{-4}$	$1.25\times 10^{-4}$
1.0	$4.54\times 10^{-5}$	$4.54\times 10^{-5}$

**Table 5:** Comparison of the exact and approximate solutions for Example 2 with other methods at $h=1/32$, $\Delta t=0.001$, $a=1$, $b=0.2$, $T=1$, $\alpha =0.1$ and $\beta =0.3$.

t	Grid point	Exact	SCM	Octic B-Spline^23^	ICNTPS^24^	EXPTPS^24^	EULTPS^24^	RMSE by SCM
$t=0.4$	0.0625	1.0459	1.0459	1.0459	1.04577	1.04577	1.04577	$6.7987\times 10^{-17}$
	0.2500	0.9414	0.9414	0.9414	0.94096	0.94096	0.94095	
	0.46875	0.8326	0.8326	0.8326	0.83265	0.83265	0.83264	
	0.56250	0.7899	0.7899	0.7899	0.79055	0.79055	0.79054	
	0.7500	0.7109	0.7109	0.7109	0.71302	0.71302	0.71302	
	0.96875	0.6288	0.6288	0.6288	0.62963	0.62963	0.62963	
$t=0.8$	0.0625	1.1330	1.1330	1.1330	1.13286	1.13286	1.13286	$6.7987\times 10^{-17}$
	0.2500	1.01981	1.01981	1.01981	1.01935	1.01935	1.01934	
	0.46875	0.9019	0.9019	0.9019	0.90224	0.90224	0.90222	
	0.56250	0.8557	0.8557	0.8557	0.85675	0.85675	0.85674	
	0.7500	0.7702	0.7702	0.7702	0.77286	0.77286	0.77285	
	0.96875	0.6811	0.6811	0.6811	0.68215	0.68215	0.68215	
$t=1$	0.0625	1.1793	1.1793	1.1793	1.17910	1.17910	1.17910	$6.7987\times 10^{-17}$
	0.2500	1.0614	1.0614	1.0614	1.06098	1.06098	1.06097	
	0.46875	0.9387	0.9387	0.9387	0.93914	0.93914	0.93913	
	0.56250	0.8906	0.8906	0.8906	0.89182	0.89182	0.89180	
	0.7500	0.8016	0.8016	0.8016	0.80450	0.80450	0.80449	
	0.96875	0.7089	0.7089	0.7089	0.71000	0.71000	0.71000

**Table 6:** Comparison between the average errors of existing methods and the Present SCM of Example 2 at $P_{e}=10\,\ \text {and} \,\ 20$.

*h*	for $P_{e}=10$				for $P_{e}=20$			
	SCM	^1^	C-order by SCM	CPU time by SCM	SCM	^1^	C-order by SCM	CPU time by SCM
1/16	$1.4695\times 10^{-10}$	$2.5028\times 10^{-5}$	6.68	0.291s	$6.1276\times 10^{-11}$	$1.9537\times 10^{-4}$	5.28	0.272s
1/32	$6.0457\times 10^{-10}$	$1.5251\times 10^{-6 }$	5.87	0.271s	$7.3774\times 10^{-11}$	$1.2147\times 10^{-5}$	5.48	0.242s
1/64	$7.5641\times 10^{-11}$	$9.3997\times 10^{-8 }$	5.77	0.279s	$3.0393\times 10^{-10}$	$7.5240\times 10^{-7}$	4.85	0.256s
1/128	$4.2917\times 10^{-11}$	$5.8331\times 10^{-9}$	5.18	0.270s	$3.7871\times 10^{-11}$	$4.6741\times 10^{-8}$	4.88	0.247s

**Table 7:** Comparison between the average errors of existing methods and the Present SCM of Example 2 at $P_{e}=100\text { and } 1000$.

*h*	for $P_{e}=100$				for $P_{e}=1000$			
	SCM	^1^	C-order by SCM	CPU time by SCM	SCM	^1^	C-order by SCM	CPU time by SCM
1/16	$8.9894\times 10^{-14}$	$1.3208\times 10^{-2}$	2.41	0.273s	$1.3562\times 10^{-19}$	$2.8826\times 10^{-1}$	0.85	0.287s
1/32	$2.8928\times 10^{-12}$	$1.2317\times 10^{-3}$	3.16	0.274s	$6.5338\times 10^{-20}$	$7.6163\times 10^{-2}$	0.83	0.262s
1/64	$1.0197\times 10^{-11}$	$8.8105\times 10^{-5}$	3.67	0.268s	$8.1057\times 10^{-18}$	$1.5195\times 10^{-2}$	1.03	0.291s
1/128	$1.4753\times 10^{-11}$	$5.6976\times 10^{-6}$	3.85	0.282s	$3.0047\times 10^{-16}$	$2.4252\times 10^{-3}$	1.88	0.257s

**Table 8:** Comparison between the exact and approximate solutions of Example 3 using the Present SCM for $\bigtriangleup t=0.05h,h=0.01, t=1,L=2$.

Grid points	for $P_{e}=1000$	
	Exact solution	Approximate solution
0.00	$-8.41 \times 10^{-1}$	$-8.41\times 10^{-1}$
0.25	$-6.81 \times 10^{-1}$	$-6.81\times 10^{-1}$
0.50	$-4.79 \times 10^{-1}$	$-4.79\times 10^{-1}$
0.75	$-2.47 \times 10^{-1}$	$-2.47\times 10^{-1}$
1.00	0.00	0.00
1.25	$2.47\times 10^{-1}$	$2.47\times 10^{-1}$
1.50	$4.79\times 10^{-1}$	$4.79\times 10^{-1}$
1.75	$6.81\times 10^{-1}$	$6.81\times 10^{-1}$
2.00	$8.41\times 10^{-1}$	$8.41\times 10^{-1}$

**Table 9:** Comparison between the absolute errors of existing methods and the Present SCM of Example 3 at $h=1/64$, $T=1$ and $P_{e}= 1000, 10{,}000 \,\ \text {and} \,\ 20{,}000$.

Grid point	$P_{e}=1000$ by SCM	^1^	^23^	$P_{e}=10{,}000$ by SCM	^1^	^23^	$P_{e}=20{,}000$ by SCM	^1^	^23^
0.25	0.0000	$1.4523\times 10^{-6}$	$9.7884\times 10^{-9}$	0.0000	$1.0552\times 10^{-6}$	$9.7898\times 10^{-9}$	0.0000	$1.0227\times 10^{-4}$	$9.7890\times 10^{-9}$
0.5	0.0000	$1.7519\times 10^{-6}$	$2.2635\times 10^{-8}$	0.0000	$4.9081\times 10^{-7}$	$2.2656\times 10^{-8}$	0.0000	$2.1975\times 10^{-4}$	$2.2657\times 10^{-8}$
0.75	0.0000	$1.8969\times 10^{-6}$	$3.7094\times 10^{-8}$	0.0000	$6.9429\times 10^{-6}$	$3.7131\times 10^{-8}$	0.0000	$3.4696\times 10^{-4}$	$3.7133\times 10^{-8}$
1	$1.45\times 10^{-60}$	$9.75\times 10^{-7}$	$4.9917\times 10^{-8}$	$7.00\times 10^{-60}$	$2.56\times 10^{-5}$	$5.0566\times 10^{-8}$	$5.15\times 10^{-59}$	$4.7082\times 10^{-4}$	$5.0639\times 10^{-8}$
1.25	0.0000	$1.0998\times 10^{-7}$	$4.9269\times 10^{-9}$	0.0000	$7.0254\times 10^{-5}$	$4.9280\times 10^{-8}$	0.0000	$6.5159\times 10^{-4}$	$4.9282\times 10^{-8}$
1.5	0.0000	$2.0490\times 10^{-7}$	$4.4664\times 10^{-8}$	0.0000	$1.5840\times 10^{-4}$	$4.4639\times 10^{-8}$	0.0000	$8.0199\times 10^{-4}$	$4.4639\times 10^{-8}$
1.75	0.0000	$2.8775\times 10^{-7}$	$3.7282\times 10^{-8}$	0.0000	$2.6866\times 10^{-4}$	$3.7221\times 10^{-8}$	0.0000	$7.4040\times 10^{-4}$	$3.7217\times 10^{-8}$

**Table 10:** Comparison between the absolute errors of existing methods and the Present SCM of Example 3 at $h=1/64$, $T=1$ and $P_{e}=0.5, 1.5 \,\ \text {and} \,\ 2.0$.

Grid point	$P_{e}=0.5$ by SCM	^2^	$P_{e}=1.50$ by SCM	^2^	$P_{e}=2.0$ by SCM	^2^
0.25	0.0000	$8.28\times 10^{-8}$	0.0000	$2.36\times 10^{-8}$	0.0000	$1.56\times 10^{-8}$
0.5	0.0000	$1.05\times 10^{-7}$	0.0000	$2.35\times 10^{-8}$	0.0000	$1.25\times 10^{-8}$
0.75	0.0000	$4.97\times 10^{-8}$	0.0000	$2.57\times 10^{-9}$	0.0000	$1.05\times 10^{-8}$
1	$6.87\times 10^{-65}$	$5.88\times 10^{-8}$	$1.09\times 10^{-63}$	$4.89\times 10^{-8}$	$1.33\times 10^{-63}$	$4.87\times 10^{-8}$
1.25	0.0000	$1.92\times 10^{-7}$	0.0000	$9.74\times 10^{-8}$	0.0000	$8.54\times 10^{-8}$
1.5	0.0000	$3.22\times 10^{-7}$	0.0000	$1.38\times 10^{-7}$	0.0000	$1.15\times 10^{-7}$
1.75	0.0000	$4.32\times 10^{-7}$	0.0000	$1.71\times 10^{-7}$	0.0000	$1.38\times 10^{-7}$

**Table 11:** Comparison between the $L_{\infty } \text { and } L_{2}$ errors of existing methods and the Present SCM of Example 3 at $h=1/10$, $\bigtriangleup t=0.1h$.

*T*	$L_\infty$ SCM	$L_2$	$L_\infty$^2^	$L_2$	$L_\infty$ method I^6^	$L_2$	$L_\infty$ method II^6^	$L_2$
0.2	0.0000	0.0000	$5.69\times 10^{-6}$	$5.07\times 10^{-6}$	$1.60\times 10^{-5}$	$1.48\times 10^{-5}$	$2.04\times 10^{-4}$	$8.64\times 10^{-5}$
0.4	0.0000	0.0000	$9.23\times 10^{-6}$	$1.14\times 10^{-5}$	$3.08\times 10^{-5}$	$2.56\times 10^{-5}$	$5.96\times 10^{-4}$	$2.63\times 10^{-4}$
0.6	0.0000	0.0000	$1.35\times 10^{-5}$	$2.01\times 10^{-5}$	$4.26\times 10^{-5}$	$3.25\times 10^{-5}$	$1.14\times 10^{-3}$	$5.52\times 10^{-4}$
0.8	0.0000	0.0000	$1.74\times 10^{-5}$	$3.05\times 10^{-5}$	$4.99\times 10^{-5}$	$3.65\times 10^{-5}$	$1.82\times 10^{-3}$	$9.56\times 10^{-4}$
1.0	0.0000	0.0000	$2.14\times 10^{-5}$	$3.94\times 10^{-5}$	$5.16\times 10^{-5}$	$4.01\times 10^{-5}$	$2.59\times 10^{-3}$	$1.47\times 10^{-3}$
5.0	0.0000	0.0000	$1.99\times 10^{-5}$	$3.62\times 10^{-5}$	$1.37\times 10^{-4}$	$1.27\times 10^{-4}$	$7.88\times 10^{-3}$	$8.90\times 10^{-3}$
10.0	$5.63\times 10^{-59}$	$5.63\times 10^{-59}$	$5.92\times 10^{-6}$	$2.09\times 10^{-5}$	$1.40\times 10^{-4}$	$1.68\times 10^{-4}$	$6.72\times 10^{-3}$	$8.81\times 10^{-3}$
20.0	$3.23\times 10^{-72}$	$3.23\times 10^{-72}$	$2.91\times 10^{-6}$	$3.34\times 10^{-5}$	$7.47\times 10^{-5}$	$9.47\times 10^{-5}$	$4.66\times 10^{-3}$	$6.02\times 10^{-3}$

**Table 12:** Comparison between the exact and approximate solutions of Example 4 using the Present SCM for $\bigtriangleup t=2h ,h=0.01, t=2, L=1$.

Grid points	Exact solution	Approximate solution
0.0	$4.11\times 10^{-3}$	$4.11\times 10^{-3}$
0.1	$6.96\times 10^{-3}$	$6.96\times 10^{-3}$
0.2	$1.15\times 10^{-2}$	$1.15\times 10^{-2}$
0.3	$1.85\times 10^{-2}$	$1.85\times 10^{-2}$
0.4	$2.91\times 10^{-2}$	$2.91\times 10^{-2}$
0.5	$4.43\times 10^{-2}$	$4.43\times 10^{-2}$
0.6	$6.53\times 10^{-2}$	$6.53\times 10^{-2}$
0.7	$9.22\times 10^{-2}$	$9.22\times 10^{-2}$
0.8	$1.23\times 10^{-1}$	$1.23\times 10^{-1}$
0.9	$1.49\times 10^{-1}$	$1.49\times 10^{-1}$
1.0	$1.53\times 10^{-1}$	$1.53\times 10^{-1}$

**Table 13:** Comparison between the absolute errors of existing methods and the Present SCM of Example 4 at $k=2h$, $T=2$ for $h=0.1 \,\ \text {and} \,\ 0.02$.

Grid point	For $h=0.1$			For $h=0.02$			
	By SCM	^1^	^2^	By SCM	^1^	^2^	^4^
0.1	0.0000	$1.62\times 10^{-4}$	$8.33\times 10^{-5}$	0.0000	$7.17\times 10^{-6}$	$2.19\times 10^{-6}$	$8.22\times 10^{-6}$
0.2	0.0000	$2.55\times 10^{-4}$	$2.30\times 10^{-4}$	0.0000	$1.10\times 10^{-5}$	$5.09\times 10^{-6}$	$2.25\times 10^{-5}$
0.3	0.0000	$3.84\times 10^{-4}$	$3.97\times 10^{-4}$	0.0000	$1.66\times 10^{-5}$	$7.97\times 10^{-6}$	$4.52\times 10^{-5}$
0.4	0.0000	$5.70\times 10^{-4}$	$6.02\times 10^{-4}$	0.0000	$2.46\times 10^{-5}$	$9.07\times 10^{-6}$	$7.77\times 10^{-5}$
0.5	$6.94\times 10^{-18}$	$8.30\times 10^{-4}$	$7.59\times 10^{-4}$	0.0000	$3.59\times 10^{-5}$	$5.18\times 10^{-6}$	$1.20\times 10^{-4}$
0.6	$1.39\times 10^{-17}$	$1.19\times 10^{-3}$	$6.52\times 10^{-4}$	0.0000	$5.16\times 10^{-5}$	$8.30\times 10^{-6}$	$1.68\times 10^{-4}$
0.7	$1.39\times 10^{-17}$	$1.67\times 10^{-3}$	$5.31\times 10^{-4}$	0.0000	$7.32\times 10^{-5}$	$3.55\times 10^{-5}$	$2.10\times 10^{-4}$
0.8	$2.78\times 10^{-17}$	$2.28\times 10^{-3}$	$7.34\times 10^{-4}$	0.0000	$1.02\times 10^{-4}$	$7.34\times 10^{-5}$	$2.22\times 10^{-4}$
0.9	$2.78\times 10^{-17}$	$3.02\times 10^{-3}$	$8.81\times 10^{-4}$	0.0000	$1.36\times 10^{-4}$	$9.37\times 10^{-5}$	$1.68\times 10^{-4}$

**Table 14:** Comparison between the absolute errors of existing methods and the Present SCM of Example 4 at $k=2h$, $T=2$ for $h=0.01$.

Grid point	By SCM	^1^	^2^	^3^	^4^	^5^
0.1	0.0000	$1.80\times 10^{-6}$	$8.97\times 10^{-7}$	$2.06\times 10^{-6}$	$2.05\times 10^{-6}$	$1.99\times 10^{-6}$
0.2	0.0000	$2.77\times 10^{-6}$	$2.28\times 10^{-6}$	$5.64\times 10^{-6}$	$5.64\times 10^{-6}$	$5.48\times 10^{-6}$
0.3	0.0000	$4.17\times 10^{-6}$	$4.12\times 10^{-6}$	$1.13\times 10^{-5}$	$1.13\times 10^{-5}$	$1.09\times 10^{-5}$
0.4	0.0000	$6.17\times 10^{-6}$	$6.18\times 10^{-6}$	$1.94\times 10^{-5}$	$1.94\times 10^{-5}$	$1.89\times 10^{-5}$
0.5	0.0000	$9.00\times 10^{-6}$	$7.82\times 10^{-6}$	$3.00\times 10^{-5}$	$3.00\times 10^{-5}$	$2.92\times 10^{-5}$
0.6	0.0000	$1.30\times 10^{-5}$	$7.90\times 10^{-6}$	$4.20\times 10^{-5}$	$4.20\times 10^{-5}$	$4.09\times 10^{-5}$
0.7	0.0000	$1.84\times 10^{-5}$	$4.95\times 10^{-6}$	$5.25\times 10^{-5}$	$5.25\times 10^{-5}$	$5.11\times 10^{-5}$
0.8	0.0000	$2.55\times 10^{-5}$	$1.74\times 10^{-6}$	$5.56\times 10^{-5}$	$5.56\times 10^{-5}$	$5.42\times 10^{-5}$
0.9	0.0000	$3.41\times 10^{-5}$	$8.78\times 10^{-6}$	$4.20\times 10^{-5}$	$4.20\times 10^{-5}$	$4.10\times 10^{-5}$

**Table 15:** Comparison between the exact and approximate solutions by SCM along with absolute errors of existing methods and SCM of Example 5 at $h=0.025$, $\bigtriangleup t=0.005$, $t=5$.

Grid point	Solutions		Absolute errors		
	Exac	By SCM	By^8^	By^7^	By SCM
3.5	0.0000000	0.0000000	$3.78\times 10^{-13}$	0	0
4.0	$1.59\times 10 ^{-5}$	$1.59\times 10 ^{-5}$	$1.02\times 10^{-9}$	0	0
4.5	$2.02\times 10^{-2}$	$2.02\times 10^{-2}$	$1.94\times 10^{-8}$	0	0
5.0	$2.18\times 10^{-1}$	$2.18\times 10^{-1}$	$1.10\times 10^{-8}$	0	0
5.5	$2.02\times 10^{-2}$	$2.02\times 10^{-2}$	$2.51\times 10^{-8}$	0	0
6.0	$1.59\times 10 ^{-5}$	$1.59\times 10 ^{-5}$	$9.45\times 10^{-10}$	0	0
6.5	0.0000000	0.0000000	$4.40\times 10^{-13}$	0	0

**Table 16:** Different errors of Examples 1–5, for different parameters.

Errors by using SCM for $h=0.01$						
Examples	Parameters	$L_2$	$L_\infty$	$Error_{AVG }$	*RMSE*	*MRE*
1	$P_e=2.5, \beta =0.25,t=2,\bigtriangleup t=2h,L=2$	$1.9230\times 10^{-16}$	$1.1102\times 10^{-16}$	$3.3307\times 10^{-18}$	$1.9230\times 10^{-17}$	$1.9230\times 10^{-16}$
2	$P_e=10, \beta =-1,t=1,\bigtriangleup t=2h,L=1$	$3.3307\times 10^{-16}$	$2.2204\times 10^{-16}$	$5.5511\times 10^{-18}$	$3.3307\times 10^{-17}$	$2.009\times 10^{-16}$
3	$P_e=1000, \beta =1,t=1,\bigtriangleup t=0.05h,L=2$	$5.7689\times 10^{-16}$	$3.3307\times 10^{-16}$	$9.9920\times 10^{-18}$	$5.7689\times 10^{-17}$	$3.9621\times 10^{-16}$
4	$P_e=10, \beta =1,t=2,\bigtriangleup t=2h,L=2$	$1.5023\times 10^{-19}$	$8.6736\times 10^{-19}$	$2.6021\times 10^{-20}$	$1.5023\times 10^{-19}$	$5.5566\times 10^{-18}$
5	$P_e=160,\beta =0.8,t=5,\bigtriangleup t=0.005,L=9$	$1.6016\times 10^{-106}$	$1.6014\times 10^{-106}$	$4.0718\times 10^{-109}$	$8.0080\times 10^{-108}$	$7.3384\times 10^{-106}$

**Table 17:** Root mean square error of Examples 1–5, for $\bigtriangleup t=3h$ by SCM.

Example	$N=16$	$N=64$	$N=100$	$N=128$
1	$5.5511\times 10^{-17}$	$2.7756\times 10^{-17}$	$2.2204\times 10^{-17}$	$1.9626\times 10^{-17}$
2	$5.4571\times 10^{-16}$	$4.7368\times 10^{-16}$	$4.0898\times 10^{-16}$	$3.6638\times 10^{-16}$
3	$8.3267\times 10^{-17}$	$4.1633\times 10^{-17}$	$3.3307\times 10^{-17}$	$2.9439\times 10^{-17}$
4	$6.5052\times 10^{-19}$	$2.1684\times 10^{-19}$	$1.7347\times 10^{-19}$	$1.5333\times 10^{-19}$
5	$1.6684\times 10^{-32}$	$2.6575\times 10^{-94}$	$1.8106\times 10^{-107}$	$1.3984\times 10^{-107}$

## Conclusions

This article explores the application of the Subdivision Scheme-based Collocation Method (SCM) to numerically solve the one-dimensional advection-diffusion equation. A conventional finite difference approach is employed to discretize the time derivatives.

The numerical results, presented in Tables 1- 17 and Figs. 2, 3, 4, 5 and 6, demonstrate the reliability of the obtained outcomes. Specifically, the exact and approximate solutions for Examples 1 and 2 with absolute errors are represented in Tables 1 and 4. Average errors of Examples 1 and 2 presented in Tables 2, 3, 6, and 7 indicate that for larger values of $P_{e}$, the current method is more efficient than the cubic $C^1$ spline-based method^1^. Table 5 represents the comparison between the exact and approximate solutions of Example 2 with the computational technique^23^, ICNTPS, EXPTPS, EULTPS methods^24^. Table 5 clearly demonstrates the accuracy of the current technique compared to the others. Exact and numerical solutions of Example 3 are listed in Table 8 which shows the accuracy of the current method. Tables 9 and 10 for Example 3 show that for smaller to larger values of $P_{e}$, our proposed method is more efficient than cubic B-Spline methods^1,2^ and the computational technique^23^. Table 11 displays the computation of errors ${L_{\infty }}$ and $L_{2}$ for different values of *T*, showing that SCM has significantly smaller errors than cubic B-Spline methods^2^ and the methods of^6^. Table 12 for Example 4 represents the accuracy of the current method. Computations of Example 4 in Tables 13 and 14 for different values of *h* indicate the current method’s accuracy for smaller values of *h* compared to the cubic B-spline^1–4^ and exponential B-spline method^5^. The data presented in Table 15 is the analysis of absolute errors of Example 5 and shows that the results obtained by SCM are very close to the exact solution when compared to the graph-theoretic polynomial collocation method^7^ and the six-order compact finite difference method^8^.

Table 16 shows the $L_\infty , L_2, Error_{AVG}, RMSE, MRE$ of Examples 1–5 for N = 100. The comparison of the *RMSE* for all examples across different values of *N* is presented in Table 17, indicating that the *RMSE* remains relatively constant as *N* increases significantly. The graphical representations from Figs. 2, 3, 4, 5 and 6 depict that the transport model by SCM converges to the exact solution very quickly for different parameters in each case.

Analysis of the data confirms the reliability, efficacy, and applicability of the current method. This technique can widely be used in fields such as fluid dynamics, heat transfer, and chemical engineering to understand and predict the behavior of different substances in various environments. In the future, we will explore this transport equation in two and three dimensions using the subdivision collocation method.

## Data Availability

The data used to support the findings of the study are available within this paper. No data was used for the research described in the article.
